# Lipid Metabolism as a Targetable Metabolic Vulnerability in Colorectal Cancer

**DOI:** 10.3390/cancers13020301

**Published:** 2021-01-15

**Authors:** Yekaterina Zaytseva

**Affiliations:** Department of Toxicology and Cancer Biology, College of Medicine, University of Kentucky, Lexington, KY 40536-0305, USA; yyzayt2@uky.edu

Colorectal cancer (CRC), the second leading cause of cancer-related deaths according to the World Health Organization, remains a substantial public health problem worldwide. Even though recent advances in early detection screenings and treatment options have reduced CRC mortality in developed nations, CRC incidence has been steadily rising globally [1]. Recently, a growing number of CRCs have been diagnosed in patients younger than 50 years of age [2,3,4,5,6]. The reasons for this alarming tendency are not well understood, but current data suggest that dietary fat uptake and obesity may be the major factors that contribute to the raising rate of early-onset CRC [7,8]. Multiple studies have corroborated the association between CRC and alterations in lipids [9,10], demonstrating that lipid metabolism has a profound impact on CRC initiation and progression and is a targetable vulnerability in this disease.

Lipids are hydrophobic macromolecules classified into eight types based on the presence of ketoacyl and isoprene groups: fatty acids (FAs), glycerolipids, glycerophospholipids, sphingolipids, sterol lipids, prenol lipids, saccharolipids and polyketide [10]. FAs are structural components of complex lipids and play a wide range of roles in human body. FAs are important substrates for energy metabolism, essential components for maintaining the structure and fluidity of all cell membranes, and building-blocks of more structurally complex lipids [11]. In addition to their role as energy substrates and structural components, lipids are also implicated in multiple functions such as signal transduction, intracellular trafficking, cell secretion, and migration [12,13,14].

Dysregulation of lipid metabolism in cancer cells, which is increasingly recognized as one of the characteristics of aggressive cancer, correlates with a poorer prognosis and shorter disease-free survival in CRC [11,15]. Increased fatty acid synthesis in 86% of aberrant crypt foci from patients with sporadic CRC or familial adenomatous polyposis also suggest its importance from early stages of colonic neoplasm development [16]. The different sphingolipid and glycerophospholipid profiles have been reported among patients with locally advanced, unresectable or metastatic CRC compared with healthy volunteers [17]. In general, higher total levels of all phospholipid classes were observed in CRC cell lines and in tumor cells isolated from CRC patients as compared to cells derived from normal mucosa [18]. Association of CRC with phospholipid signatures was confirmed by analysis of fresh frozen sections of CRC tissue and adjacent healthy mucosa from 12 patients using MALDI mass spectrometry, a novel technique which allows simultaneous localization and quantification of biomolecules in different histological regions [19].

FAs acquired by cancer cells, either by endogenous synthesis or by exogenous uptake, are rapidly incorporated into cellular triglycerides [20], which are the main form of lipid storage and major source of energy in the human organism. Excessive triglycerides and cholesterol in cancer cells are stored in lipid droplets, and high numbers of lipid droplets have been reported in CRC as compared to normal colonic tissues [21,22]. CRC stem cells also contain higher amounts of lipid droplets than their differentiated counterparts, as revealed by Raman spectroscopy imaging, and the number of lipid droplets directly correlates with well-accepted CRC stem cell markers such as CD133 and Wnt pathway activity [23]. Interestingly, lipid droplets are used not only to maintain lipid homoeostasis and prevent lipotoxicity, but also to provide a valuable source of adenosine triphosphate (ATP) and nicotinamide adenine dinucleotide phosphate (NADPH) during conditions of metabolic stress [24].

Serum lipid profiling also represents an attractive avenue for novel cancer biomarker discovery. Multiple studies have demonstrated that profiling of lipids in the blood can distinguished CRC patients from healthy individuals and can be a diagnostic tool for early-stage CRC [25]. Analysis of 234 serum samples from three groups of patients (66 with CRC, 76 with polyps, and 92 healthy controls) demonstrated that CRC patients can be distinguished from healthy controls and individuals with colorectal polyps based on their serum levels of linoleic and α-linolenic acid [26]. In another study, CRC patients presented with nearly 50% lower serum concentrations of γ-linolenic acid than healthy controls, which lead the authors to proposed it as a biomarker for CRC risk [25]. Analysis of plasma from CRC patients shows that the level of triglycerides is increased in plasma of advanced-stage versus early-stage CRC patients [27].

Taken together, delineating the differences in lipid metabolism and lipid profiles between CRC patients and healthy individuals can provide not only potential biomarkers and diagnostic tools, but can also identify potential therapeutic targets for CRC.

In mammalian cells, FAs can either be obtained from diet or synthesized de novo from carbohydrate precursors. In contrast to normal epithelial cells, the majority of FAs in malignant cells are derived from de novo lipogenesis regardless of the availability of extracellular lipids [11,28]. Fatty acid synthase (FASN), a key enzyme of lipid biosynthesis, catalyzes the synthesis of palmitoyl-CoA from acetyl-CoA, malonyl-CoA and NADPH [29]. Palmitate can be elongated and desaturated through the activity of stearoyl-CoA desaturase and elongation of very long chain fatty acid proteins to produce additional FA species including stearate and oleate which can subsequently be used for production of more complex lipids [24]. FASN is significantly upregulated in CRC and is associated with advanced stages of CRC and CRC metastasis [15,30,31]. Upregulation of FASN promotes tumorigenic properties of CRC cells via upregulation of multiple oncogenic pathways such as Wnt, HER2, PI3K/Akt, AMPK/mTOR, and cMET [15,31,32,33,34]. The increased reliance of cancer cells on FAs not only sustains their rapid proliferative rate and enhances migration and invasion, but also provides an essential energy source during conditions of metabolic stress [10]. Indeed, increased activity of FASN is associated with β-oxidation of endogenous lipids and promotion of cellular respiration in CRC cells [22]. De novo lipogenesis is also associated with enhanced saturation of membrane lipids in CRC cells, making the cells less susceptible to free radicals and penetration of chemotherapeutic agents [35]. Preclinical studies have shown that inhibition of FASN and de novo palmitate synthesis induces apoptosis in tumor cells by remodeling cell membranes, inhibiting oncogenic signaling pathways, and reprogramming gene expression [33,36]. In vivo studies show that shRNA-mediated inhibition of FASN significantly reduces lung and hepatic metastases in nude mice and inhibits angiogenesis in an orthotopic CRC model [15,30]. Consistently, pharmacological inhibition of FASN suppresses liver metastasis in mice [37]. Targeting other enzymes within the de novo FA synthesis pathway has also been evaluated, including ATP citrate lyase (ACLY) and acetyl-CoA carboxylase (ACC). ACLY, a first-step rate-limiting enzyme for de novo lipogenesis, is upregulated and promotes metastasis and mediates drug resistance in CRC [38,39]. Inhibition of ACC induces apoptosis in CRC cells [40]. Taken together, current data strongly support that de novo lipogenesis significantly contributes to progression of CRC and CRC metastasis and targeting this pathway is a promising therapeutic strategy in CRC.

While most tumors exhibit a shift toward FA synthesis, cancer cells can also scavenge lipids from their environment by upregulating various FA-uptake mechanisms including FA transporters [11]. The most characterized of FA transporters include CD36, also known as fatty acid translocase, fatty acid transport protein families (FATPs), and plasma membrane fatty acid-binding proteins (FABPs). The reports on CD36 in CRC are inconsistent and its role in this disease requires further clarification [41,42,43,44]. Interestingly, upregulation of CD36 has been shown to be a potential compensatory mechanism that drives resistance to TVB-3664, a novel inhibitor of FASN, which potentially suggests that simultaneous inhibition of FA uptake and synthesis would be required to achieve a desirable anticancer effect in CRC [43]. Alongside CD36, FATPs and FABPs regulate long- and very long-chain FA uptake and transport to the various compartments within the cell. FABP4 overexpression increases FA transport to enhance energy and lipid metabolism, and activates the AKT pathway and epithelial-mesenchymal transition to promote migration and invasion of CRC cells [45]. High expression of FABP5 is also associated with cell growth and metastatic potential of CRC cells [46]. Interestingly, the expression of FABP6 is also higher in primary CRC and adenomas than in normal epithelium, but is dramatically decreased in lymph node metastases, suggesting that FABP6 may play an important role in early carcinogenesis [47]. Taken together, the uptake and scavenging of extracellular FAs not only allows metabolic flexibility, but also provides an important compensatory mechanism for cancer cells to sustain their lipid demands under conditions of metabolic stress.

The relationship between obesity and colorectal cancer risk is well established [48,49,50,51,52]. Epidemiological studies demonstrate that high fat diet and obesity increase the risk of CRC by 1.5–2.0-fold, with obesity-associated colon cancer accounting for 14–35% of the total incidence [53]. In the cohort study that followed 121,050 adults for 26 years, intake of proinflammatory diets including high fat diet was associated with a significantly higher risk of developing CRC in both men and women [54]. Furthermore, abdominal visceral adipose tissue volume contributes to the development and growth of colorectal adenomas, and it is a better predictor for risk of colorectal adenomas than body mass index or waist circumference in both sexes [55]. The interconnection between adipose tissue and development of CRC can be explained if adipose tissue functions as an endocrine system that secretes growth factors, cytokines and free FAs following lipolysis [24]. Also, adipose tissue is the main reservoir of triglycerides, capable of releasing them into the bloodstream, and thus, it may influence the lipid profiles of various tissues [10]. Interactions between adipose compartment and tumorigenesis have growing interest among researchers. Delineating the mechanisms driving interactions between CRC cells and adipocytes has provided fundamental insights into how obesity contributes to tumor initiation and progression [24].

Our understanding of the precise role of lipid metabolism in CRC is still very limited. Given that lipids regulate very diverse cellular processes and influence a wide range of tumorigenic steps in cancer development, progression and metastasis, there is substantial clinical interest in developing therapies to target FA metabolism. Even though no lipid metabolism therapies are currently used in clinic for CRC, analysis of lipid metabolism in preclinical models and cancer patients strongly supports lipid metabolism as a targetable vulnerability in CRC and better insight into FA-mediated metabolic disturbances can define new directions in diagnosis and treatment of this disease. Improvements in conventional methods of lipid detection and quantification, as well as development of new spectroscopic, imaging and data processing methods have greatly advanced our ability to delineate the differences in lipid profiles and lipid metabolism [55] and have opened new opportunities for drug development.
